# Spider egg sacs reveal how pockets of air can be used to conserve water

**DOI:** 10.1242/jeb.250298

**Published:** 2025-07-14

**Authors:** Katherine Q. Karkosiak, Ravi Z. Schwartz, Hunter King, Todd A. Blackledge

**Affiliations:** ^1^ Department of Biology and Integrated Bioscience Program, The University of Akron, Akron, OH 44325, USA; ^2^ Department of Physics, and Center for Computational and Integrative Biology, Rutgers University–Camden, Camden, NJ 08103, USA

**Keywords:** Porous materials, Nonwoven materials, Water homeostasis, Water balance, Diffusion, Membrane

## Abstract

Controlling water transport across surfaces is essential for all living organisms. Spider egg sacs are multifunctional membranes that protect eggs and spiderlings from the external environment. Past research gives conflicting results about whether these mats of silk fibers reduce evaporation of water in part because the diffusive resistance of any membrane cannot be measured independently of the system in which it is studied. We developed a model to describe water vapor transport across porous surfaces that includes the important roles of the gap space underneath the membrane and the boundary layer on the outside of the membrane in controlling water vapor flux, in addition to the relative impermeability of the membrane itself. The model accurately predicts diffusive resistance of a variety of synthetic surfaces from empirical studies, as well as the egg sacs of the black widow *Latrodectus hesperus* and the garden spider *Argiope aurantia*. We show that ‘typical’ spider egg sac membranes offer surprisingly low diffusive resistance to water because they are highly porous at microscopic scales. However, silk egg sacs still play key roles in controlling water loss by preserving and defining an internal region of stagnant air that often dominates the diffusive resistance of the whole system. Our model provides a tool to explore diverse spider egg sac geometries, but can also be adopted to fit a variety of systems to facilitate comparison and engineering of diffusive resistance across membranes.

## Teaser

**Highlighted Article:** Modeling water loss across porous surfaces shows that silk egg sacs offer low diffusive resistance but still control water loss by preserving internal stagnant air.

## INTRODUCTION

It is essential for all living organisms to control water transport, from osmosis across plasma membranes at the cellular level to transpiration across plant leaves and diffusion across cuticles at the whole organism level. Specializations that might control water transport across these surfaces, such as hydrophobic coatings or pores, also influence other key processes including gas exchange and thermoregulation (e.g. evaporative cooling) (Costa et al., 2013). Moreover, surfaces that may appear ‘opaque’ when viewed at larger scales are often heterogeneous at smaller spatial scales. Plant leaves contain stomata (Bange, 1953), eggshells are penetrated by pores (Rahn et al., 1987), and even cellular membranes contain active and passive transport channels (Verkman et al., 1996). Understanding the mechanisms by which these barriers influence water loss is therefore essential, but complex.

**Fig. 1.**
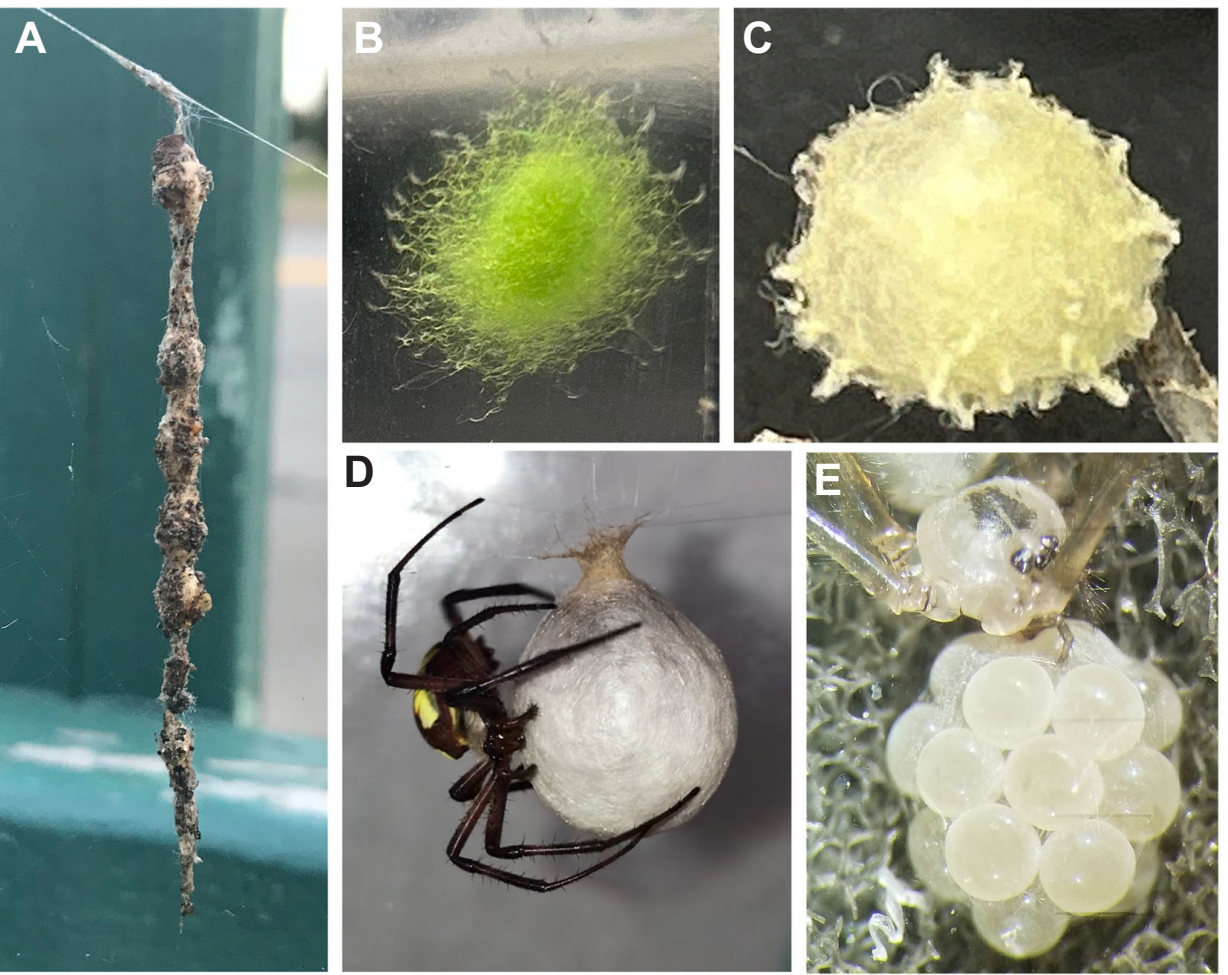
**Variation in the morphology of spider egg sacs.** (A) Egg sacs of *Cyclosa* sp. (Araneae: Araneidae) are covered in a thick layer of silk and debris. (B) The egg sac of *Gasteracantha* sp. (Araneae: Araneidae) is covered in thick loops of silk and colored (likely for camouflage). (C) The egg sac of *Latrodectus geometricus* (Araneae: Theridiidae) shows numerous silk protuberances. (D) The egg sac of *Argiope aurantia* (Araneae: Araneidae) is covered by a thick, smooth layer of silk. (E) The egg sac of *Pholcus manueli* (Araneae: Pholcidae) consists of only a few silk threads surrounding the eggs (photo credit: Alex Salazar, University of Miami, Oxford, OH, USA, July 2022).

Many organisms undergo sensitive developmental stages early in life or during metamorphosis inside external structures such as cocoons and eggshells that provide protection from predators and environmental hazards such as thermal fluctuations and desiccation. For example, all spiders spin silk egg sacs around their eggs. Although egg sacs vary greatly in morphology across species (Fig. 1), they are all generally non-woven fibrous mats that are presumed to play a key role in maintaining water balance for the developing embryos (Anderson, 1973), particularly because spider egg sacs lack the serosal membrane found on insect eggs that prevents desiccation (Jacobs et al., 2013). However, studies investigating the role of silk egg sacs in limiting water loss for developing spiders yield inconsistent results. Schaefer (1976) found increased survival of *Floronia bucculenta* (Araneae: Linyphiidae) at lower humidity when the egg sac was left intact versus when the egg sac was removed, whereas Austin and Anderson (1978) found no effect of the egg sac on the survival of *Trichonephila edulis* (Araneae: Nephilidae) at varying humidity. Hieber (1992a) measured the water loss, hatching success, molting success and spiderling survival of *Mecynogea lemniscata* (Araneae: Araneidae) and *Argiope aurantia* (Araneae: Araneidae) and found no effects of the egg sac for *A. aurantia*, but increased water loss and reduced spiderling survival for *M. lemniscata* at low humidity. To try to isolate the effect of the egg sac silk layer, Opell (1984) measured water loss through swatches of Uloboridae egg sac silk sealed to capillary tubes filled with water and kept in a desiccator at low humidity, and found that the silk layer significantly slowed the water loss from the tubes at rates that differed among species. Although these studies span diverse species with potentially varying sensitivity to water loss, the seemingly contradictory conclusions of these studies reflect not just differences in methodology, but also conceptual differences in what water balance of the whole ‘system’ means, and how individual components such as egg chorion, silk membrane of the egg sac, or egg sac geometry and structure play distinct roles.

**Fig. 2.**
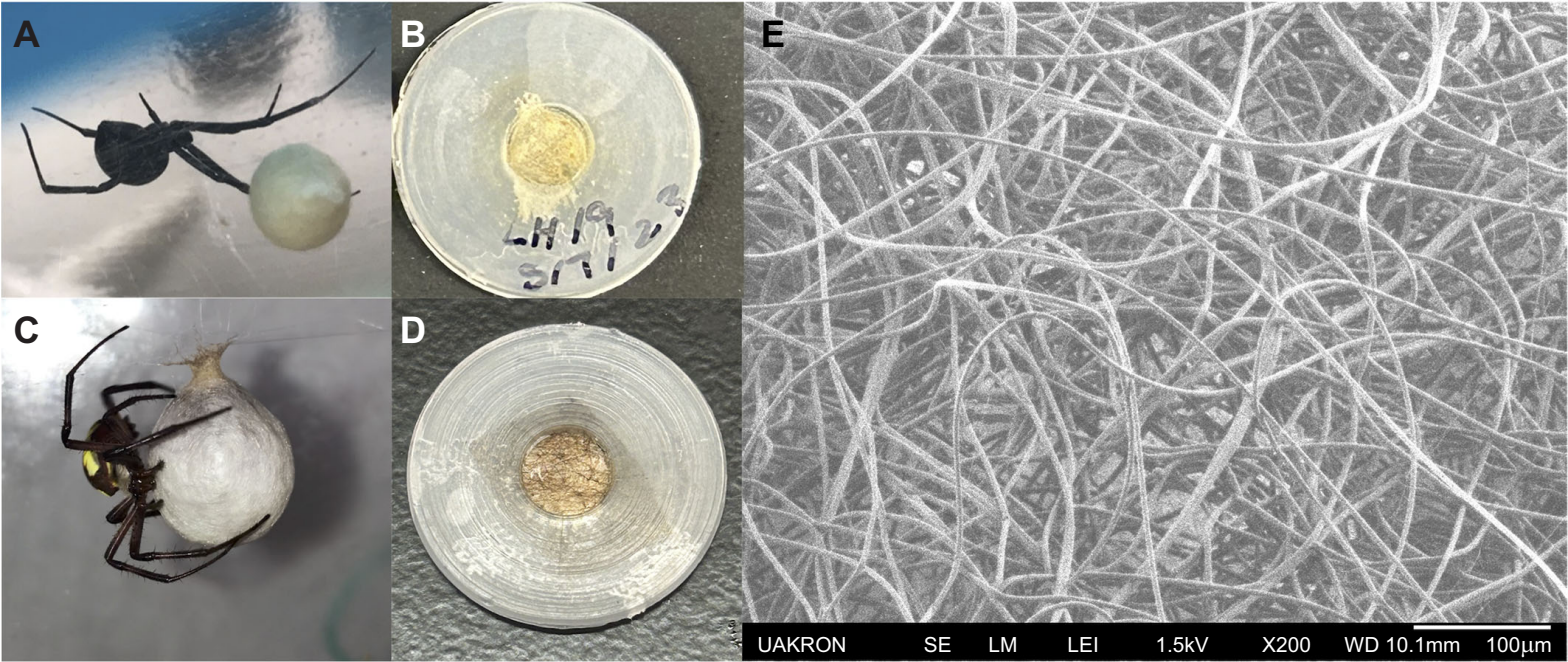
**Egg sac microstructure and sample preparation.** (A) Female western black widow, *Latrodectus hesperus*, with egg sac and (C) female yellow garden spider, *Argiope aurantia*, building an egg sac. (B,D) Swatches of silk membrane taken from (B) *L. hesperus* egg sac and (D) *A. aurantia* egg sac glued between two plastic washers with 5 mm central opening diameter. (E) Scanning electron microscope image of exterior of *L. hesperus* egg sac, showing the porous microstructure and arrangement of silk fibers.

Here, we present a quantitative model to characterize how thin, porous barriers such as the silk membranes of spider egg sacs (Fig. 2E) mediate water transport via diffusion through the air. The model predicts diffusive flux across the whole system through independently measurable structural properties and geometry by applying Fick's law consecutively to each distinct region of the system from the most interior surface (with local water vapor concentration *c_i_*_n_) to the ambient air (with vapor concentration *c*_out_). The total diffusive flux Ψ (mass of water leaving the surface per unit time) is driven by the difference in concentration of water molecules (Δ*c*=*c*_in_–*c*_out_):
(1)

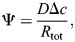
where *D* is the diffusion coefficient for water vapor in air (with dimensions area/time) and *R*_tot_ is the total resistance to water flux where each individual resistance *R_n_* depends on the geometry of the corresponding region (*R*_tot_=*R*_1_+*R*_2_+*R*_3_+…).

**Fig. 3.**
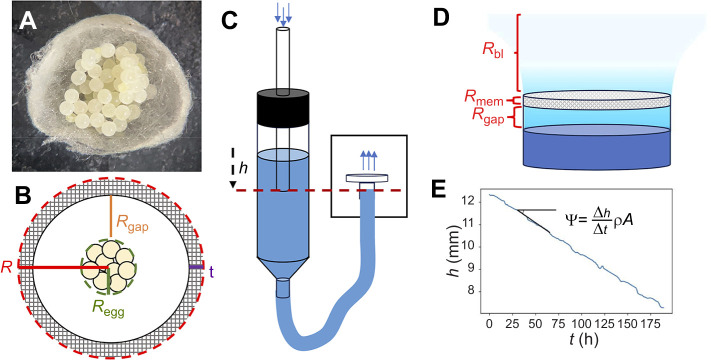
**Empirical and theoretical modeling of water loss across spider egg sacs.** (A) Cross-sectional cut through a western black widow (*Latrodectus hesperus*) spider egg sac showing the central egg mass, the gap space and the outer silk layer. (B) Simplified diagram of an egg sac showing the three main resistance components: silk membrane resistance (*R*_mem_), gap space resistance (*R*_gap_) and exterior boundary layer resistance (*R*_bl_). (C) Mariotte bottle where the height of the water underneath the sample of silk egg sac (*h*) is held constant and determined by the height of the reservoir's inner tube. As water diffuses across the silk, air enters the reservoir to maintain pressure and a constant *R*_gap_ is maintained. (D) Resistance components for a cylindrical tube. (E) Flux is calculated from the slope of the height of the water in the reservoir over time, times density ρ and cross-sectional area *A*.

In the case of the typical spider egg sac system (see Fig. 3A), a simplified idealization of water flux from the egg mass to the outside environment (see Fig. 3B) includes three key components described below. Their derivations (see Supplementary Materials and Methods 1, Fig. S1) follow from a framework proposed by Bange (1953) to model the flux of water through the pores of plant leaves.

### Theoretical model and experimental setup

*R*_gap_ is defined by the empty space between the egg mass (with average radius *r*_egg_) and the inner surface of the silk membrane of the egg sac (defined as the average outer radius, *r*, minus the thickness, *t*, of the silk membrane):
(2)

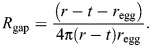
Although no solid mass obstructs the random motion of water molecules (ignoring the presence of flocculent silk in some egg sacs), the thickness of still air nevertheless limits net water flux. Importantly, there is significant variation among spider species in how closely appressed the silk cover of the egg sac is to the egg mass.

Resistance of the silk cover of the egg sac, *R*_mem_, is defined by the morphology of the silk membrane itself:
(3)

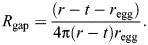


A key realization here is that visually opaque silk membranes are full of microscopic air gaps between silk strands that allow for diffusive exchange across the membrane. Egg sac membrane resistance is therefore proportional to the solid volume fraction, where φ is the porosity (percentage of open air space in the material), with the assumption that diffusion through the silk itself is negligible. Because molecules must diffuse around solid elements in a circuitous path between inside and outside, an effective thickness of the membrane τ*t* is used, where τ is the tortuosity, a measure of the wiggliness of paths partially obstructed by solid elements through the membrane (expressed as function of porosity τ=1–ln(φ) for disordered fibers (Shen and Chen, 2007) and *t* is the membrane's actual thickness. Although tortuosity can significantly increase the path length of molecules diffusing across the thickness of a densely packed mat of fibers, it plays a negligible role toward high porosity, in which paths become generally unobstructed.

*R*_bl_ is the boundary layer between the outside surface of the silk membrane and the distance at which moisture concentration reaches ambient values:
(4)

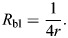


The thickness of the outside boundary layer determines its resistance similar to that of the thickness of the empty layer (Eqn 3). The boundary layer, at low flow rates, is determined primarily by the size of the object. Notably, the boundary layer is completely outside the silk egg sac, yet still provides a potentially key resistance to total flux which still depends on the egg sac geometry.

Because water molecules must pass successively through each region to leave the system, the total resistance to diffusive flux from the egg mass (*R*_tot_) then becomes:
(5)

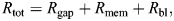
and questions regarding how the design of spider egg sacs regulates water loss relate to the overall and relative magnitudes of these three terms. Importantly, this model illustrates how previous studies on desiccation resistance of spider egg sac membranes cannot directly infer the resistance of the silk egg sac membrane itself to diffusion by measuring the performance of the whole system (*R*_tot_) because of the varying influences of egg mass shape, morphology of the exterior of the egg sac, and even the external environment on total resistance. Although these three terms are sufficient for modeling a generic egg sac, additional resistance terms can be added to account for more unique egg sac geometries. Indeed, our model provides a vital tool to extract the contribution of each individual component of the system.

Here, we employ the model in experimental investigation of the competing roles of the egg sac silk properties and geometry in preventing water loss using Mariotte bottles (McCarthy, 1934) (see Fig. 3C) instead of sealed tubes. This simple mechanical trick keeps *R*_gap_ constant by maintaining ambient pressure in an outside reservoir of water that constantly refills the tube covered by the egg sac membrane as water evaporates. Then, total flux is calculated using the constant rate of decreasing water level in the outside reservoir (Fig. 3D), from which an experimental value of *R*_tot_ is obtained using Eqn 1.

We performed two types of experiments. First, we validated the model by comparing the resistances of synthetic membranes that vary over broad ranges of membrane porosity and morphology (e.g. thick versus thin and mostly porous versus mostly open). These samples were deliberately fabricated to easily characterize the input parameters of the model, to directly compare the model's predictions with experimental results. Then we used the same experimental design to compare the resistances of natural egg sac samples from two species of spiders with open and closed controls. We performed these experiments in two different conditions – low and high air flow – to further probe the importance of the boundary layer compared with the internal components for total resistance of egg sacs to water vapor flux. Finally, we explored the design principles that might be at play in the enormous interspecific variation found in spider egg sac construction among the world’s over 53,000 described species of spiders (World Spider Catalog, http://wsc.nmbe.ch).

## MATERIALS AND METHODS

### Diffusion experiments using the Mariotte bottle design

To measure the total flux of water through membranes of spider egg sac silks and synthetic porous materials, we modified a method by Opell (1984) to include a Mariotte bottle. Opell (1984) measured evaporation over time for capillary tubes covered by silk egg sacs and found significant variation among species in rate of water loss (*R*_tot_). However, Opell (1984) could not directly measure the effect of the silk membrane itself (*R*_mem_) because the measurements of total water loss were conflated by the varying influence of *R*_gap_ as water levels dropped at different rates through the experiment. Our Mariotte bottle design (Fig. 3C) measured water loss from tubes that were constantly refilled from an external reservoir to maintain a constant distance between the evaporating surface and the sample (*h* in Fig. 3C), and in turn keeping gap resistance (*R*_gap_) constant. Experiments with synthetic materials versus egg sac material had slightly different design, but both followed the same general design. The sample of material was glued over the top of a cylindrical chamber with the evaporating liquid level closely below the sample, and a tube at the bottom of the chamber was connected to an outside reservoir of the liquid to act as a Mariotte bottle and maintain pressure inside of the system equal to the atmospheric pressure while keeping the water level constant on the sample side (Fig. 3C). As water diffused out through the sample, the water level below the sample stayed the same, but the water level in the reservoir decreased. The rate of water loss was measured by recording the change in the water level in the reservoir (Fig. 3E) over the duration of the experiment using time-lapse camera images. Although the exact dimensions of a particular setup determined *R*_gap_, that value remained constant throughout the experiment.

### Resistance measurement with synthetic membranes

To first validate the model, we measured the flux through a variety of synthetic porous membranes that varied significantly in thickness and porosity (see Supplementary Materials and Methods 2). These membranes were adhered over 6×1 cm Petri dishes using Super Glue™, a method adapted from Zwieniecki et al. (2016). The membranes included thin acrylic discs with straight holes, metal wire meshes, and electrospun nano-fiber mats with varying porosity and thickness values (outlined in Table S1). Many of the synthetic materials were fabricated with predetermined geometries with discrete, straight pores, allowing us to easily calculate their porosity, tortuosity and theoretical *R*_tot_. For the more complex membranes, porosity (φ) was calculated either via their volume fraction or, in the case of the electrospun material (Fig. S2), via liquid intrusion porosimetry. For mesh geometries (freely overlapping cylinders), we calculated their tortuosity (τ) as a function of porosity. For the straight pores, τ=1, by definition, according to the equation τ=1−ln(φ) (Shen and Chen, 2007).

To expedite these trials and to avoid the effects of variation in ambient humidity, we used pure ethanol as our diffusing vapor. Only the diffusibility constant, *D*, for ethanol differed from corresponding experiments with water. The Petri dishes were placed inside a large partially enclosed box (15.24×15.24×30.48 cm) to act as a baffle against ambient airflow from lab ventilation. We measured the flux, or loss of ethanol, in the reservoir using time-lapse images taken with a Nikon^®^ 5300 DSLR camera (Nikon, Melville, NY, USA) at 60 min intervals for approximately 3 days. From the flux, we calculated the material's resistance to diffusion. We compared the fit between the total flux predicted by the model with that measured in the empirical experiments using linear regression implemented in Statistica v.10 (StatSoft, OK, USA), calculating *r*^2^ as a measure of fit.

### Measuring water flux across spider egg sacs

We measured the water loss through egg sac swatches from two spider species: the black and yellow garden spider, *Argiope aurantia* Lucas 1833 (5 swatches from 3 egg sacs), and the western black widow, *Latrodectus hesperus* Chamberlin & Ivie 1935 (7 swatches from 6 egg sacs). *Argiope aurantia* were collected from the Bath Nature Preserve, Summit County, OH, USA, and *L. hesperus* were purchased from Bugs of America. Egg sacs were cut open using dissection scissors and a swatch from the egg sac outer layer was glued over the 5 mm diameter opening of a plastic washer (0.9 mm thick) using GUGUYeah^®^ UV light curing glue (Shenzhen Fuyada Industry, Shenzhen, China). The washer with the sample was then glued to the end of a silicone tube attached to the reservoir, which was made from a modified 10 ml syringe. The sample washer at the end of each reservoir was placed into a desiccator containing Drierite^®^ (W. A. Hammond Drierite, Xenia, OH, USA) crystals to keep the ambient air conditions relatively constant. Up to five samples were placed into the desiccator at a time for each trial, typically one of each type of spider egg sac, an open negative control tube and a Parafilm-covered positive control tube. The volume of water loss was measured using a Nikon^®^ D5100 camera to capture time-lapse photos of the external reservoir every 10 h for 50–70 h. Water volume loss was then found using Fiji (Schindelin et al., 2012) to measure the change in water height inside the 10 ml syringes. The initial and final temperature and humidity measurements inside the desiccator were taken using a thermometer/hygrometer (Traceable^®^ Products, Webster, TX, USA).

We conducted two different types of experiments by varying air flow in the desiccator to manipulate the magnitude of the boundary layer (*R*_bl_). ‘No fan’ experiments were conducted with ambient still air in the desiccator, allowing a greater influence of the boundary layer on total resistance of water diffusion. ‘Fan’ experiments were conducted with turbulent air continuously moving across the sample tubes by enclosing an 80×80×25 mm CPU computer fan (Wderair, Shenzhen, China) facing perpendicularly across the samples (flow of 74.08 m^3^ h^−1^). The moving air greatly reduced the boundary layer and therefore largely removed *R*_bl_ from the total resistance in the ‘fan’ trials.

### Egg sac data analysis

We compared the average measured flux, or rate of water loss, through swatches from each sample type in each condition (wind, no wind), where each swatch counted as a sample (*L. hesperus*: *n*_nofan_=9, *n*_fan_=7; *A. aurantia*: *n*_nofan_=3, *n*_fan_=5; open tubes with no coverings: *n*_nofan_=5, *n*_fan_=11; and Parafilm coverings: *n*_nofan_=5, *n*_fan_=12), at 50 h in each condition (wind, no wind) using a two-way ANOVA test implemented in JMP^®^ Pro, Version 16 (SAS Institute). Then we calculated the total resistance to the diffusive flux of each sample type (using Eqn 1) to better compare the sample types by controlling for changes in humidity or temperature between trials. Because both temperature and humidity likely varied within the 50 h of each experiment, we made two separate sets of calculations – one for the parameters that maximized flux and the other for the parameters that minimized flux.

### Confocal image analysis of egg sac membranes

We measured both the thickness and porosity of egg sacs. We obtained confocal image *z*-stacks of cut sections of the egg sac layers using a Zeiss LSM 510 META confocal microscope at 10× magnification and ZEN system software (Carl Zeiss Microscopy, Jena, Germany). We used the fluorescein isocyanate setting and an HFT 405/488/543/633 laser with a range of additional settings to obtain an image stack for one egg sac from each species. Using ImageJ software (Schneider et al., 2012) with Fiji (Schindelin et al., 2012), we calculated the porosity as the percentage of pixel space not filled with silk in each image slice found using the Threshold feature to select the number of pixels taken up by the fibers, and subtracted this from the total number of pixels in the image (see Tables S2 and S3). This was repeated for each image in the stack, and the mean porosity percentage of all images in the stack was used to find the sample average. To find the thickness of the sample, we multiplied the step size by the number of steps in the stack (number of images minus one).

## RESULTS

**Fig. 4.**
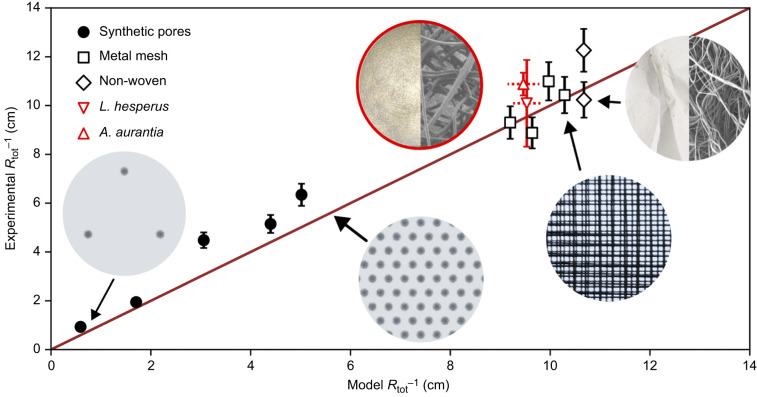
**Model validation using synthetic membranes and comparison to spider egg sacs.** Measured values of evaporative loss versus predictions based on the model. The *y*-axis is presented as conductance (*R*_tot_^–1^) rather than resistance to better show the linear relationship. Error bars indicated one standard deviation. The solid line represents perfect agreement between experiment and prediction [(*R*_tot_^–1^)_exp_=(*R*_tot_^–1^)_pred_]. Linear regression found close agreement: d.f.=1,9, *P*<0.000001, *R*^2^=0.96. The red markers show the spider egg sacs. The dotted horizontal lines indicate the minimum and maximum conductance predicted by the model given variation in experimental parameters; the vertical lines indicate one standard deviation from the empirical experiments. Although both egg sacs and electrospun samples (furthest right) look opaque to the eye (left half of insets), they are actually quite porous (∼93% and 97% average porosity for *L. hesperus* and *A. aurantia*, respectively) at the microscopic scale (right half of insets) and therefore highly conductive to diffusive loss compared with the other materials.

First, we tested the model using synthetic samples. Fig. 4 compares measured versus modelled total diffusive resistance for a range of porous membrane systems. The comparison is made using conductance, which is simply resistance^−1^, because conductance more clearly shows the linear relationship. The total resistances are calculated according to Eqns 2–5, including the gap and boundary layer resistances. The samples range from high resistance Petri dish covers with small, sparse holes (lower left), to mostly open ∼mm-scale mesh screens, to low resistance, high porosity electrospun fiber mats (upper right).

Two observations stand out. First, the model accurately predicts measured resistances across the broad range of sample structures from impermeable solids with straight holes to nonwoven fabrics (linear regression d.f.=1,9, *P*<0.000001, *R*^2^=0.96; Table S1). Second, the resistance provided by different membrane morphologies does not necessarily agree with our intuitive expectations. In particular, the visibly open mesh screens (Fig. 4, inset in center) provide more resistance than the distinctly opaque fiber mats (Fig. 4, far right inset). The model and experimental results convey an important fact that our eyes confuse: microscopic pores are still large compared with water molecules – the more porous the material, the more empty space water molecules have to traverse, almost regardless of whether the space is organized into many microscopic pores or fewer large pores.

Using values of thickness and porosity measured from samples of egg sac used in our experiment (see Materials and Methods), we calculated the predicted *R*_tot_ for silk egg sacs (red markers in Fig. 4). Owing to their similarly high porosity and thickness, egg sacs predict just slightly higher resistance than the electrospun fiber mats. This presents a first indication that the silk membrane's resistance to diffusion may not be the primary mechanism by which spider egg sacs control water loss.

**Fig. 5.**
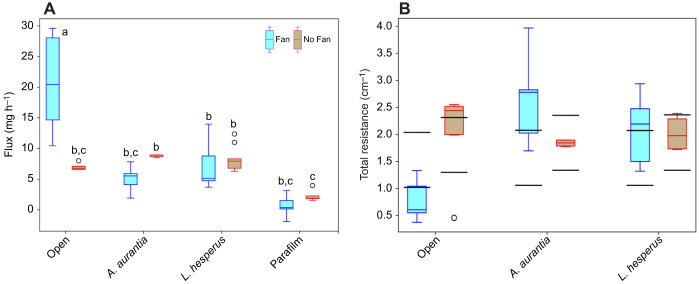
**Water loss across spider egg sac membranes.** (A) Measured average flux through natural egg sac samples covering cylindrical tubes, with and without the presence of a fan in the evaporation chamber. Open tubes lost significantly more water in moving air than in still air. In still air, egg sacs performed no better than open tubes, indicating the negligible role of *R*_mem_, but also showed no increased water loss when wind was added to the system, demonstrating the crucial role of the silk membranes in preserving the system's overall geometric resistance. (B) Corresponding total resistances calculated from Eqn 1, in comparison with those predicted by the theoretical model, based on sample geometry alone (black bars show bounding values based on experimental uncertainties). The shift between ‘fan’ and ‘no fan’ predicted bounds comes from setting *R*_bl_=0 for windy conditions. Parafilm provides a strong diffusive barrier and acts as a negative control, for which a measured flux is likely due to diffusion through the Mariotte bottle's air-inlet tube.

Next, we experimentally tested water loss rates through two different egg sac materials, adapting the Mariotte bottle design for smaller sample sizes. Fig. 5 displays rate of total water loss (flux) over time and the theoretical diffusive resistance for egg sac samples of two species of spiders, *L. hesperus* (*n*_nofan_=9, *n*_fan_=7) and *A. aurantia* (*n*_nofan_=3, *n*_fan_=5), open tubes without a covering (*n*_nofan_=5, *n*_fan_=11) and tubes with a Parafilm covering (*n*_nofan_=5, *n*_fan_=12). Comparisons were made in both still air and wind blowing across the membranes inside the desiccator. In still conditions, evaporation in tubes covered by spider egg sacs was greater than Parafilm-covered tubes and instead indistinguishable from open tubes. In windy conditions, this relationship switched, and open tubes exposed to wind lost significantly more water than all other sample types, whereas tubes covered by egg sacs showed no difference to their performance in still conditions (two-way ANOVA; fan presence *F*=2.2730, *P*=0.1053; sample type *F*=25.0086, *P*<0.0001, sample type×fan presence *F*=14.5585, *P*<0.0001). Although flux values appeared to decrease in the fan trials, the difference was not significant and can be explained by the higher water losses driving faster increases in humidity in the chamber throughout the experiments.

Because experiments with natural egg sac samples used water (rather than ethanol) as the diffusing molecule, the raw measurement of flux depended strongly on variation in the ambient humidity, through its relationship with Δ*c* in Eqn 1. This confounding effect of the experimental sample with environmental factors determining raw flux data is resolved by calculating Δ*c* from measured relative humidity, and expressing results in *R*_tot_ in Fig. 5B. One-to-one comparisons with the model predictions can then be made without reference to environmental conditions. The black bars in Fig. 5B represent the maximum and minimum possible calculated resistance given the variation in relative humidity during the experiment and uncertainty in the measured thickness of the air gap *h* between sample and meniscus. A notable exception is the open tube, where the model overpredicts the resistance in the presence of wind. In this case, disagreement with the prediction highlights a design element: lacking even a highly porous membrane, the air flow likely penetrates the tube, causing advective loss of moisture not captured by the model, which depends on slow, purely diffusive flux in *R*_gap_. Comparison of resistance terms in the model indicates that the spider egg sac's role in maintaining that gap of still air near the eggs is more important than its material role in directly resisting loss via diffusion across the silk (*R*_mem_).

**Fig. 6.**
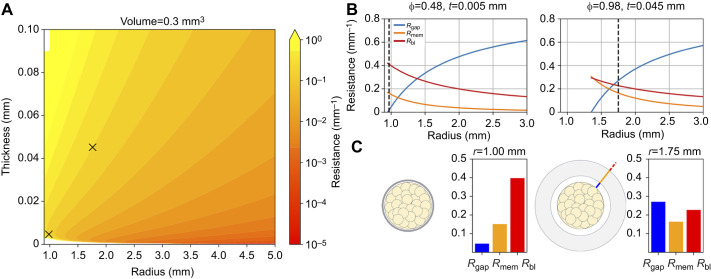
**Modeling of variation in egg sac geometry.** (A) Contour plot shows the effects of variation in egg sac design (egg sac outer radius, *x*-axis; egg sac thickness, *y*-axis) on the total resistance (color bar) for fixed volume of silk. Two arbitrarily chosen points, indicated by ×, show how different pairs of design variables give the same total resistance. (B) Variation in resistance as a given volume of silk with constant porosity and tortuosity is plotted as function of increasing outer radius of the egg sac. Note the sudden increase in dominance of *R*_gap_ as the egg sac increases past a critical diameter. Vertical blue dashed line indicates the radius plotted as×in A. (C) Visual representations of the two designs of equal resistance show that the same volume of silk can be applied as dense, closely appressed coverings or more distant, fluffier coverings to reach the same end. Next to each representation is a bar graph indicating the corresponding role of individual resistance terms in each design.

The two species of spider measured in our experiment represent a very tiny fraction of the rich variation in egg sac size and morphology among spiders. However, we can use the model to explore how the geometrical and material variation of egg sacs are predicted to contribute to resistance of water loss for a larger range of egg sac geometries. Fig. 6 illustrates how varying thickness, external radius, tortuosity and porosity values contribute to total resistance and how each of the three individual components vary. The heat map in Fig. 6A shows total resistance values as function of membrane thickness *t* and external radius *r*, calculated using Eqns 2–4, while holding total silk thread volume constant. In other words, we modeled how a spider's ‘choice’ to deposit a finite amount of silk in different egg sac shapes influences desiccation resistance. We identified two arbitrarily chosen points along a contour line of constant total resistance, to represent two sets of design parameters that provide the same outcome – one point represents a thin (*t*=0.005 mm), dense (φ=0.36) silk ‘shell’ while the other represents a thick (*t*=0.06 mm), highly porous (φ=0.95) ‘fluffy’ silk cover. Fig. 6B then shows how resistance changes as those membrane morphologies are used to construct bigger versus smaller egg sacs. Here, total volume of silk necessarily varies and the blue lines indicate the specific sizes corresponding to the same volume of silk (0.3 mm^3^) shown in Fig. 6C. Fig. 6C shows the corresponding individual resistances (*R*_gap_, *R*_mem_ and *R*_bl_) evaluated in the bar graph next to a schematic of the geometry of each solution. Perhaps non-intuitively, the thin shell design derives resistance more from the boundary layer than from the silk directly blocking diffusion owing to the egg sac's small surface area. In contrast, the fluffy design retains moisture mostly owing to the significant role of *R*_gap_. It is worth noting that the magnitude of *R*_mem_ in the fluffy case results mostly from the difficulty in diffusing through such a large region of air rather than from direct obstruction by its extremely sparse solid silk elements.

## DISCUSSION

Controlling the exchange of matter and energy with the environment is a fundamental and complex challenge for organisms. For instance, structural adaptations that may be beneficial for water regulation may interfere with competing needs for thermoregulation or respiration (Costa et al., 2013). The silk egg sacs produced by spiders define the developmental environment of eggs and young spiderlings. The morphology of egg sacs varies enormously across the diversity of spiders (Fig. 1), likely reflecting important functional interactions in the exchange of gases, heat and water with the external environment. Although initial expectations might suggest that many egg sacs act as simple barriers to diffusion of water, we show that the silk membrane creates three regions contributing to the resistance to water loss: the gap space, the membrane and the boundary layer. Further, the performance of the whole egg sac system often depends more strongly on where the silk membrane is placed relative to the egg mass than on the actual diffusive resistance caused by the silk fibers in the membrane. The visually opaque silk mats from the egg sacs of two different species of spider in our study provided almost no direct resistance to diffusion in still air (Fig. 5), but still played a key role in determining the resistances of the internal (*R*_gap_) and external (*R*_bl_) regions through which water must additionally pass. Natural systems often need to respond to varying flow conditions and many egg sacs are exposed in the environment and are therefore expected to face variation in air flow. We found that the silk membranes contributed no more resistance than open tubes in our experiment when there was no wind, but that the silk barrier prevented a significant increase in water flux once wind was added to the system (Fig. 4). This demonstrates the silk's role in preserving the diffusive resistance of *R*_gap_ by preventing advective flows into the interior regions of egg sacs, even in situations where the silk itself does little to directly inhibit water molecules from passing through its thickness. The sometimes-dominant role of the empty spaces (*R*_gap_ and *R*_bl_), compared with the silk membrane itself (*R*_mem_), helps explain the divergent conclusions of past studies on desiccation resistance of spider egg sacs because inferences about the role of the silk membrane itself are often determined by the geometry of the experimental setup. Our experiments were designed to validate the model, which is then used to predict performance of natural designs of spider egg sacs. Because the geometry of the samples does not match that of the natural cases, the fluxes should not be directly compared. Our experiment likely represents something close to worst case scenario for spiders where eggs would desiccate completely in a few hours to days owing to the extreme gradient in relative humidity and complete loss of boundary layer in the fan treatment. However, the findings are very relevant to more natural conditions because of the enormous range of time that eggs and spiderlings spend inside of the egg sacs. In our study species alone, time inside of the egg sac ranges widely: it is approximately 2 weeks for *L. hesperus* (Kaston, 1970) and, in temperate environments, *A. aurantia* can spend the entire winter inside of the egg sac, emerging in the spring (Hieber, 1985). Our model allows us to extract the contribution of each resistive component in the whole system to tease apart meaningful results from experiments with diverse geometries, even when they do not resemble natural egg sacs.

This new understanding of the role of geometry also reframes our understanding of the evolution of diverse egg sac morphologies to include egg sac shape and size as functional mechanisms for controlling water vapor loss. By modeling the resistances of whole egg sacs, we find that larger egg sacs (e.g. moving silk resources into a thinner membrane further away from the eggs) provide greater resistance to water loss owing to increased resistance of *R*_gap_. This gap resistance can be so great that the silk membrane itself plays an insignificant role in reducing water loss. This could allow reduced investment in silk material or selection for other functions such as puncture resistance against parasitoids (Hieber, 1992a,b; Austin and Anderson, 1978). In contrast, smaller egg sacs may rely more heavily on the silk membrane owing to a smaller gap resistance. As egg size and egg sac size are both likely constrained by spider body size, our model reveals the potential for multiple egg sac morphologies to have the same total desiccation resistance produced by variations in the geometry and silk placement around the eggs that manage the threat of water loss (Fig. 6B).

The model also proposes new hypotheses for the external morphologies of egg sacs. The egg sac membrane, particularly the three-dimensionality of its outer surface, defines a boundary of water vapor gradient surrounding the silk, which is at least partially protected from airflow. This boundary layer is important because, although some species shelter their egg sacs with leaves or crevices, many species of spider produce egg sacs that are exposed to the elements in nature. This finding also suggests a hypothesis for the functional significance of projections, such as the spikes seen on brown widow (*Latrodectus geometricus*) egg sacs, the looped fibers around mimetid egg sacs, and protruding silk threads, such as seen in species of long-jawed orb weavers (*Tetragnatha* sp.), because these may effectively increase the boundary layer resistance in a manner similar to eyelashes in mammals, which protect the eye from drying (Amador et al., 2015). Clearly, water loss is not the only factor affecting the evolution of spider egg sac structure, such that changes in geometry and silk structures related to water balance will also influence other aspects of egg sac function such as thermal maintenance, gas exchange and physical protection against parasitoids. These relationships could be antagonistic or synergistic. But the principles developed in this model can be applied to other concepts such as heat transfer to help understand those relationships. For instance, heat conductance is increased by denser packing of silk fibers and increased random orientation of fibers. The necessarily smaller gap distance between eggs and silk this would require (for a given investment of silk) should increase both water loss and heat transfer. Conversely, more gap spaces and air space between pores is beneficial for insulation but could be largely neutral for water loss in morphologies when *R*_gap_ dominates. The interplay between heat and water vapor transfer should be further explored. For instance, metabolic heating from the eggs or external heating from the sun causing the eggs to rise in temperature differently than surrounding structures should, in principle, cause some natural convection in the internal region, an effect which is not accounted for in the model. Such exploration may also reveal critical thresholds in surface topologies beyond which functional gains are no longer realized, such as is seen between percent coverage of stomata in leaves and gas exchange (Zwieniecki et al., 2016). Although our model does not explore the other diverse functions of egg sacs (thermoregulation, parasitoid/predation resistance, etc.), it identifies likely axes of variation in egg sac morphology that might be acted upon by natural selection. Using this model, we can better understand the influence of egg sac morphology on water loss and accurately predict performance of each component, which can allow for more complex evolutionary studies of egg sac morphology based on ecology, behavior and life history.

The model developed in this paper provides a generalizable approach for investigating water loss across porous membranes for various systems in biology, such as the resistance of other egg-encasing structures or cocoons, transpiration across integuments, the boundary layer effect of plant hairs or spines (Schreuder et al., 2001), or even leaf-enclosed habitats produced by a variety of leaf-rolling insects (e.g. *Manduca sexta* eggs; Woods, 2010). The model can even be applied in industrial settings, such as for the development or application of textiles, electrospun mats, porous membranes and other synthetic surfaces. By applying a ‘whole-system’ approach to the material and geometric parameters contributing to resistance of water loss across barriers, we can better understand the influence and accurately predict performance of each component.

## Supplementary Material

10.1242/jexbio.250298_sup1 Supplementary information
