# Supplementary material for: Spider egg sacs reveal how pockets of air can be used to conserve water
Source: J Exp Biol. 2025 Jul 14;228(13):jeb250298. doi: 10.1242/jeb.250298 (PMC12315551; doi:10.1242/jeb.250298)
Supplement: Supplementary information [file jexbio-228-250298-s1.pdf]

Supplementary Materials and Methods 1

Cylindrical pore model for experimental results

Brown and Escombe (1900) proposed a model for calculating the flux through stomata based on Fick's law by assuming they function as straight holes in a septum separating a liquid from the open air. Their general expression for static diffusion is:

$$Q = k\frac{p-p_1}{L}At \tag{1}$$

Where Q is the amount of matter diffusing through the pore, k is the diffusivity constant, $p-p_1$ is the concentration at opposite ends of the pore, L is the depth of the pore, A is the area cross-section, and t is time. This becomes recognizable as analogous to Ohm's law:

$$e = f(v-v')\frac{A}{L}t \text{ or } \quad \frac{e}{t} = \frac{(v-v')}{r} \tag{2}$$

With e being the quantity of electricity flowing through a conductor in time t, f being the conductivity constant, and $v-v'$ as the electrical potential. This equation is more commonly known as $I = \frac{V}{R}$ where R is the resistance. Bange (1953) uses this to simplify the equation into:

$$\frac{Q}{t} = i = \frac{(c-c')}{R} \tag{3}$$

Where i represents diffusion rate, $c-c'$ is the total concentration difference, and R is the total resistance and is equal to the sum of all resistances much like resistors in series. According to Bange R_{tot} is comprised of three resistors in series.
Resistance of the pores:

$$r_p = \frac{l}{k\pi s_1^2 N} \tag{4}$$

Here s_1 represents the radius of the pores and N corresponds to the total number of pores in the septum with a depth l.

Resistance of the micro-vapor cup:

$$r_{mi} = \frac{1}{k4s_1N} \tag{5}$$

And the resistance of the macro-vapor cup:

$$r_{ma} = \frac{1}{k4s_2} \tag{6}$$

Here s_2 is the radius of the septum.

Journal of Experimental Biology • Supplementary information

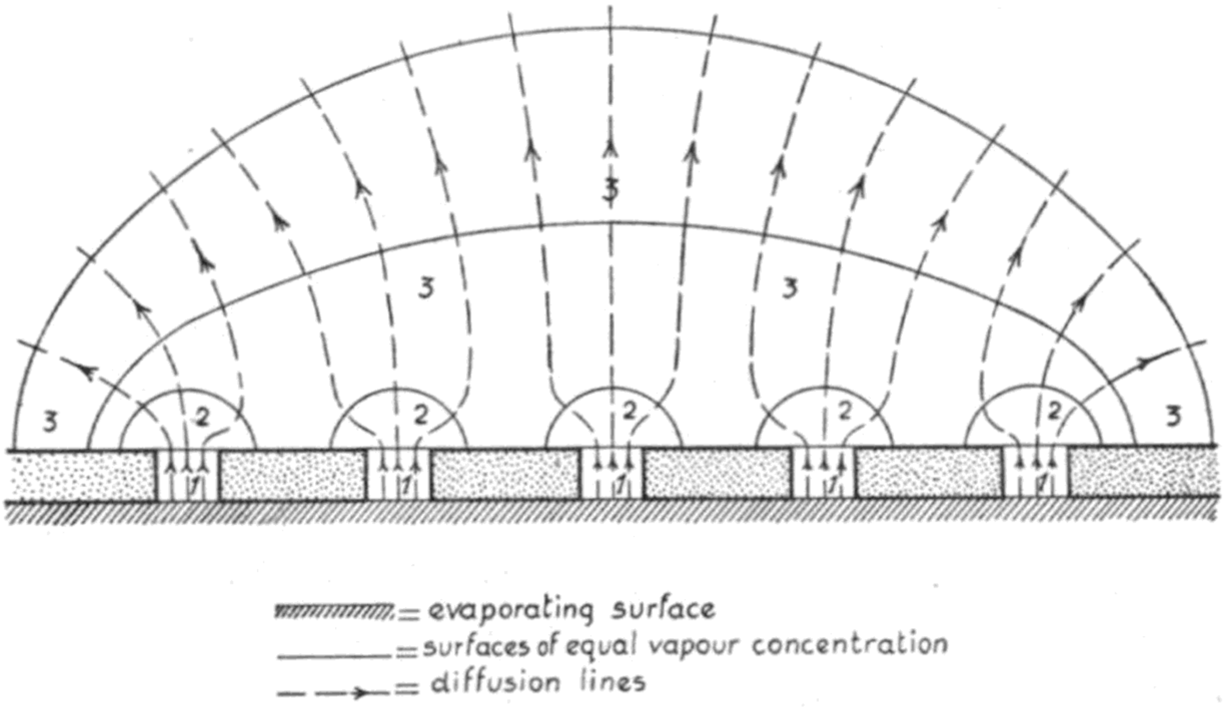

Fig. S1. Pathway of diffusion passing through the different resistive sections. Section 1 represents the pore resistance. Section 2 represents the micro-vapor cup resistance. Section 3 represents the macro-vapor cup resistance (Bange 1953, with permission).

For the purposes of this paper the constant k is moved outside of the resistance term to define resistance only as a function of geometry. Additionally, when considering spider egg sacs and our experiments with high porosity synthetic membranes, the resistance from the micro-vapor cup becomes negligible and a resistance inside the egg sac is added. Thus, the three resistive domains become: R_{gap}, R_{mem}, and R_{bl}.

$$R_{gap} = \frac{h}{\pi r^2} \quad (7)$$

Where h is the distance from the water to the membrane and r is the radius of the membrane.

$$R_{mem} = \frac{\tau t}{\phi 2\pi r^2} \quad (8)$$

Where τ is the tortuosity and equal to $1 - ln(\phi)$ for a random array of freely overlapping cylinders (Shen and Chen 2007) t is the membrane thickness and ϕ is the membrane porosity.

$$R_{bl} = \frac{1}{4r} \quad (9)$$

It should be noted that R_{bl} diminishes quickly as flow is introduced and can be considered negligible with even low to moderate wind speeds.

Journal of Experimental Biology • Supplementary information

When calculating the resistance and accounting for the spherical shape of a traditional egg sac we amended the previous equations by again following ohm's law, but this time, understanding the system as a point charge in a multiple layered conductive sphere. This way by integrating the resistances over the surface of their respective layers we can model the theoretical resistance of that layer. A similar approach could be used for more complex, non-spherical geometries.

$$R = \rho \frac{L}{A} \tag{10}$$

$$dR = \rho \frac{dr}{4\pi r^2} \tag{11}$$

$$\int \; dR = \int_{R_1}^{R_2} \frac{\rho \, dr}{4\pi r^2} \tag{12}$$

$$R = \frac{\rho}{4\pi}\left[\frac{1}{R_1} - \frac{1}{R_2}\right] \tag{13}$$

$$R = \frac{\rho}{4\pi}\left[\frac{R_2 - R_1}{R_1 \cdot R_2}\right] \tag{14}$$

Where ρ is 1 for R_{gap} and R_{bl}, but $\frac{t}{\phi}$ for R_{mem}. ϕ is the porosity.

Now

$$R_{gap} = \frac{(r - t - r_{egg})}{4\pi(r - t)r_{egg}} \tag{15}$$

Where r_{egg} is the distance from the center of the egg sac to the outside of the egg clutch, and r is the distance from the center to the outside of the egg sac.

$$R_m = \frac{\tau t}{\phi 4\pi r^2} \tag{16}$$

$$R_{bl} = \frac{fr - r}{4\pi f r^2} \tag{17}$$

Here f is a scaling factor with upper and lower bounds 2 and 1. At f=1, flow is negligible, and at f = 2, the boundary layer thickness is estimated as equal to the sphere's radius, following the argument cited above for the cylindrical case.

Supplementary Materials and Methods 2

Experimentation with Synthetic Porous Materials

Various materials were used in the synthetic pore experiments. The straight pore membranes were made from 2mm thick acrylic disks with a 3cm radius. Each disk had a number of uniform holes with a radius of 0.5mm cut out of them via a laser engraver. The number of pores included 19, 61, 127, 217, 271. The different forms of metal wire screens and meshes were used for more tortuous synthetic membranes. These included window screen folded over both two and three times the synthetic spider like material was electrospun Poly(N-isopropylacrylamide) or PNIPAm.

Journal of Experimental Biology • Supplementary information

Table S1. Synthetic materials used for diffusion experiments and their porosity, thickness and number of holes.

Parameter tested	Material	Porosity	Thickness	Number of holes
Discrete pores	Acrylic	0.0055	2mm	19
Discrete pores	Acrylic	0.018	2mm	61
Discrete pores	Acrylic	0.037	2mm	127
Discrete pores	Acrylic	0.060	2mm	217
Discrete pores	Acrylic	0.078	2mm	271
Tortuous materials	Stainless steel mesh	0.87	0.33 cm	N/A
Tortuous materials	Galvanized steel super spall particle filter	0.14	0.08 cm	N/A
Similar to spider silk	PNIPAm electrospun material	0.98	0.0006 cm	N/A
Tortuous materials	Metal screen folded 2x	0.80	0.136 cm	N/A
Tortuous materials	Metal screen folded 3x	0.80	0.189 cm	N/A

The synthetic spider like material was created via electrospinning. Electrospinning is a technique that allows the generation of polymer fibers with a nanometer scale diameter (Formhals 1934). Electrospun fibers were used because they exhibit properties such as interconnected ultrafine fibrous structures, high surface to volume ratio tortuosity, high porosity and particularly the uniform morphology of the fibers (Ibrahim and Klingner 2020) figure SI1. The porosity of these electrospun materials was measured using Liquid intrusion porosimetry (LIP) where the empty volume in a material is replaced with a known liquid (in this case water) and the total mass is compared to the base material without the liquid (Liu and Lannutti 2001).

Journal of Experimental Biology • Supplementary information

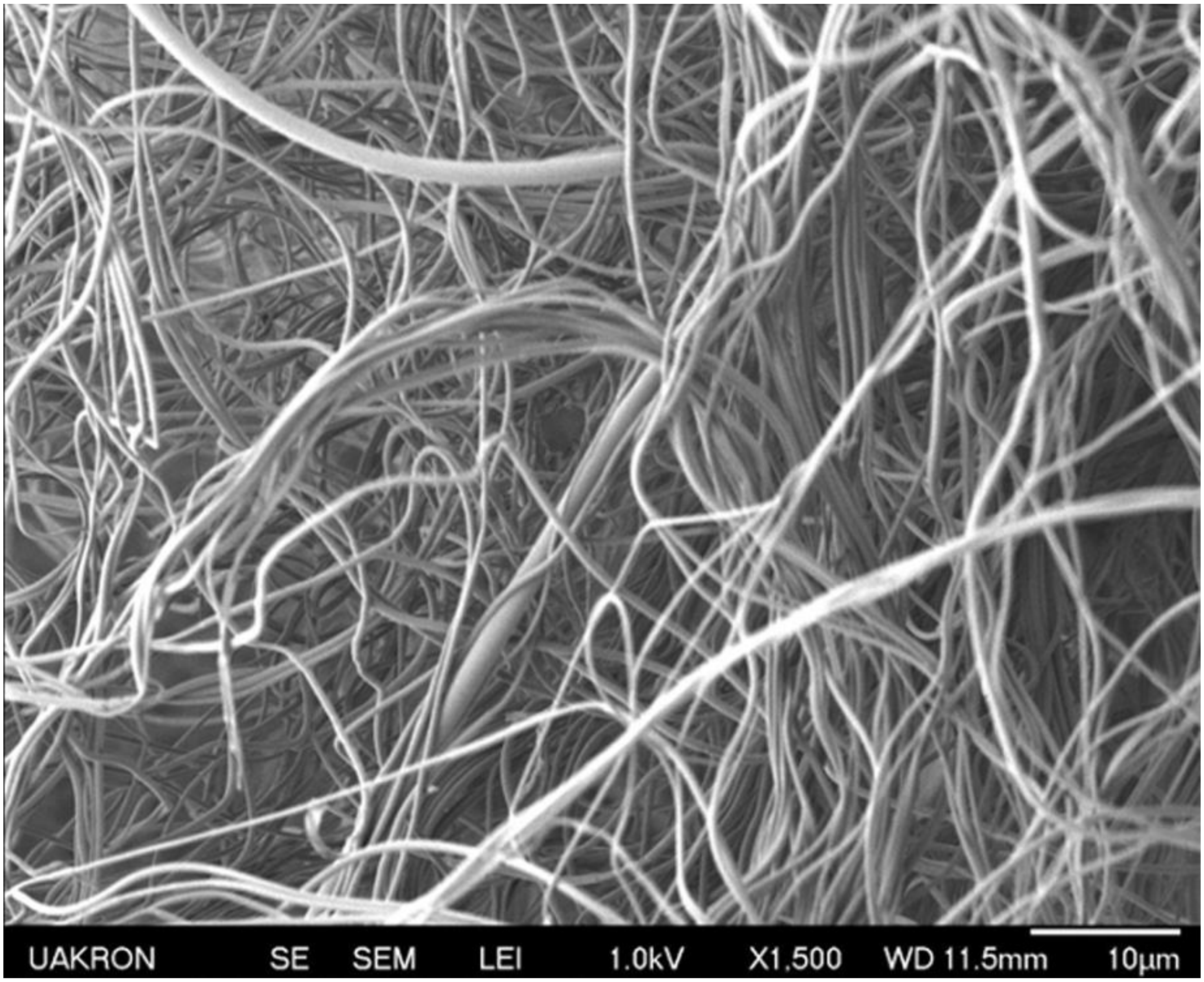

Fig. S2. SEM of electrospun 10% w/v PNIPAm (Nepal 2023).

Table S2. Values of confocal image parameters for L. hesperus and A. aurantia image stacks.

Image parameters	*L. hesperus*	*A. aurantia*
Pixel size (μm)	0.82	0.41
Step Size (μm)	13.26	14.27
Image total area (μm^2)	705062.5024	176265.6256
Threshold (range pixel value)	166-255	39-255

Table S3. Porosity values measured for each image in the L. hesperus and A. aurantia confocal image stacks.

	L. hesperus	*A. aurantia*
**Image Number**	**Porosity (%)**	**Porosity (%)**
Image 1	96.511	97.039
Image 2	95.938	95.698
Image 3	95.509	95.818
Image 4	95.017	97.201
Image 5	94.429	97.455
Image 6	93.813	97.542
Image 7	93.524	97.49
Image 8	93.24	97.049
Image 9	93.173	96.242
Image 10	92.915	96.405
Image 11	92.466	96.742
Image 12	91.501	97.034
Image 13	90.439	96.764
Image 14	89.575	96.911
Image 15	89.394	97.484
Image 16	90.06	97.516
Image 17	91.098	97.63
Image 18	92.137	97.231
Image 19	93.216	96.693
Image 20	94.189	95.638
Image 21	94.879	94.746
Image 22	95.472	95.113
Image 23	96.031	95.696
Image 24	96.458	96.165
Image 25		96.304
Image 26		96.733
Image 27		97.172
Image 28		97.664
**mean%**	**93.374**	**96.685**
**Thickness (μm)**	**304.980**	**385.290**
**total volume (μm^3)**	215029961.982	67913382.887
**volume empty space (μm^3)**	200782793.468	65661932.971

Journal of Experimental Biology • Supplementary information

References

Bange, G.G.J. (1953). On the Quantitative Explanation of Stomatal Transpiration. *Acta botanica neerlandica*, *2*(3), **255–297.**

Brown H, Escombe F. (1900). Static diffusion of gases and liquids in relation to the assimilation of carbon and translocation in plants. Philosophical Transactions of the Royal Society of London. Series B, Biological Sciences 193: **223–291**.

Formhals, A. (1934). Process and apparatus for preparing artificial threads us patent specification, 1975504.

Ibrahim, H.M., & Klingner, A. (2020). A review on electrospun polymeric nanofibers: Production parameters and potential applications. *Polymer Testing, 90*, 106647.

Liu, Y. & Lannutti, J. (2021). Characterization of electrospun porosities: current techniques.

Nepal, S. (2023). Sorbent Based Atmospheric Vapor Harvesting: Energy Delivery To Material Choice. University of Akron, Doctoral dissertation. OhioLINK Electronic Theses and Dissertations Center, http://rave.ohiolink.edu/etdc/view?acc_num=akron1690543153814168

Shen, L. and Chen, Z. (2007). Critical review of the impact of tortuosity on diffusion. Chemical Engineering Science, 62(14):**3748–3755**.

Journal of Experimental Biology • Supplementary information
